# The subcortical role in executive functions: Neural mechanisms of executive inhibition in the flanker task

**DOI:** 10.3758/s13415-024-01215-7

**Published:** 2024-09-05

**Authors:** Nofar Strommer, Hadas Okon-Singer, Shai Gabay

**Affiliations:** Department of Psychology, University of Haifa, Haifa, Israel

**Keywords:** Cognitive control, Visual cortex, Neural network, Attention

## Abstract

While executive functions (EFs) have traditionally been linked to the cerebral cortex, our understanding of EFs has evolved with increasing evidence pointing to the involvement of cortico-subcortical networks. Despite the importance of investigating EFs within this broader context, the functional contributions of subcortical regions to these processes remain largely unexplored. This study addresses this gap by specifically examining the involvement of subcortical regions in executive inhibition, as measured by the classic Eriksen flanker task. In this study, we used a stereoscope to differentiate between subcortical (monocular) and cortical (mostly binocular) visual pathways in EF processes. Our findings indicate that monocular visual pathways play a crucial role in representing executive conflict, which necessitates cortical involvement. The persistence of a monoptic advantage in conflict representation highlights the substantial contribution of subcortical regions to these executive processes. This exploration of subcortical involvement in executive inhibition provides valuable insights into the intricate relationships between cortical and subcortical regions in EFs.

## Introduction

Executive functions (EFs) are an umbrella term encompassing many cognitive-based skills involved in mental control and self-regulation (Chan et al., 2008; Diamond, 2013; Malenka et al., 2009). Executive functions generally refer to “higher-level” cognitive functions involved in the control and regulation of “lower-level” processes and goal-directed, future-oriented behavior (Alvarez & Emory, 2006). The most widely accepted model that provides a clear taxonomy of EFs designates three main cognitive abilities: *inhibition*—deliberate overriding of dominant or prepotent responses; *updating*—constant monitoring and rapid addition/deletion of working memory contents; and *shifting*—adaptably switching between tasks or mental sets (Miyake et al., 2000). The current study was designed to examine subcortical involvement in EFs, particularly in executive inhibition, which has previously been shown to have common cognitive and neural mechanisms with other EF processes, such as updating (Booth et al., 2014; McNab et al., 2008).

Over the past two decades, extensive research has focused on operationalizing and measuring EFs because of their integral role in everyday cognitive processes, ranging from basic automatic tasks to complex social functions (Alvarez & Emory, 2006; Diamond, 2013; Lezak et al., 2012). Executive functions have traditionally been linked to prefrontal activity; "[pre-]frontal lobe deficit" has been observed in individuals performing poorly on EF tests (Stuss & Alexander, 2000; Duke & Kaszniak, 2000; Duffy & Campbell, 2001). While the prefrontal cortex (PFC) has an established role in monitoring EFs, contemporary perspectives underscore the involvement of dynamic and flexible networks comprising of both cortical and subcortical regions.

Neuroimaging studies have contributed significantly to uncovering the neural mechanisms underlying EFs, which include not only cortical but also subcortical regions (Friedman & Robbins, 2022; Bellebaum & Daum, 2007; Botvinick et al., 2001; Carter et al., 1999; Cohen et al., 2000; D’Esposito & Postle, 2002; Yarkoni et al., 2005). Meta-analyses, such as those conducted by Neindam et al. (2012), have noted increased activity in a common cognitive control network during EF tasks, encompassing the DLPFC, frontopolar cortex, orbitofrontal cortex, and anterior cingulate. Noteworthy activations extend to superior and inferior parietal, occipital, temporal cortex, and subcortical areas, including the caudate, putamen, thalamus, and cerebellum. Moreover, recent perspectives emphasize the importance of investigating the relationship between cortical and subcortical regions, and elucidating the cortical-subcortical circuitry (Menon & D’Esposito, 2021; Hazy et al., 2007; Pauli et al., 2016).

While we recognize the significance of examining EFs within the broader context of cortico-subcortical networks, it is important to note that the functional contributions of subcortical regions to these cognitive processes have been comparatively underexplored in the existing literature. Only a few studies have focused on the specific role that subcortical regions play in EFs. For instance, recent inquiries by Saban et al. (2018) and Peskin et al. (2020) have demonstrated the involvement of subcortical mechanisms in executive inhibition process, as assessed by the Stroop interference effect. To delve into subcortical involvement, the researchers employed a stereoscope, which allows for a distinction between the contributions of monocular and binocular visual pathways, a detailed explanation of which is provided below. In brief, by manipulating the visual information presented to each eye separately, we can examine the involvement of monocularly segregated subcortical regions of the visual processing stream in a specific cognitive function. Saban et al.’s (2018) primary findings revealed an interference effect only when both the color patch and the word were presented to the same eye, as opposed to when each was presented to a different eye. These outcomes strongly support the case for subcortical involvement in driving the Stroop interference effect. As a result, the authors concluded that—in line with previous research—subcortical regions have a functional influence on executive functions, operating beyond the mere conveyance of information to the cortex.

As noted, the majority of the research dedicated to exploring neural structures underlying EFs, particularly that which focuses on executive inhibition processes, has emphasized cortical mechanisms. Despite this emphasis, there is compelling evidence pointing to the involvement of subcortical regions in these cognitive processes. In order to discern the roles played by cortical and subcortical structures in EFs, it is imperative to systematically investigate their distinct contributions to these processes. The current study addresses this gap in the literature through the use of a stereoscope method. Employing a stereoscope facilitates the differentiation between monocular and binocular visual pathways, providing a unique opportunity to isolate subcortical involvement in EFs. The primary goal of this study is to comprehensively examine the functional contribution of subcortical regions to EF processes, specifically in the domains of executive inhibition. Our research seeks to delineate the functional roles of subcortical structures in distinct EFs, thereby contributing to a more nuanced understanding of the neural substrates involved in these cognitive processes.

We employed a well-established EF task, namely the Eriksen flanker task (Eriksen & Eriksen, 1974), to investigate the executive inhibition process, which was adapted for stereoscopic presentation (details outlined below). Our central hypothesis posits a discernible functional role for subcortical regions, as measured through monocular visual pathways, in executive inhibition. We expect variations in the pattern of results between monoptic and dichoptic presentation of visual stimuli, reflecting the contribution of subcortical regions to executive inhibition.

## Examination of subcortical involvement in executive inhibition using the Eriksen flanker task

A variation of the Eriksen flanker task (Eriksen & Eriksen, 1974) was adapted to the stereoscope (detailed below) to examine participants’ response inhibition abilities and their neural mechanisms.

### Participants

Thirty-six participants (12 males; 23.6 ± 3.6 years) consented to participate. All participants had normal or corrected-to-normal visual acuity (i.e., only corrective lenses, no spectacles). Previous studies examining the manipulation of stereoscopic presentation revealed large effects sizes (i.e., ηp^2^ = 0.2) (Saban et al., 2018). A power analysis in G*power indicated that with a sample size of 22 participants we could detect similar effect size (f = 0.5) with power of 0.98. Hence, our sample size was well above the required to identify such effects. Participants were reimbursed for their time either via payment (30 NIS) or course credit. The institutional review board of the University of Haifa approved the experimental protocol (approval 032/16).

### Stimuli

**Fig. 1:**
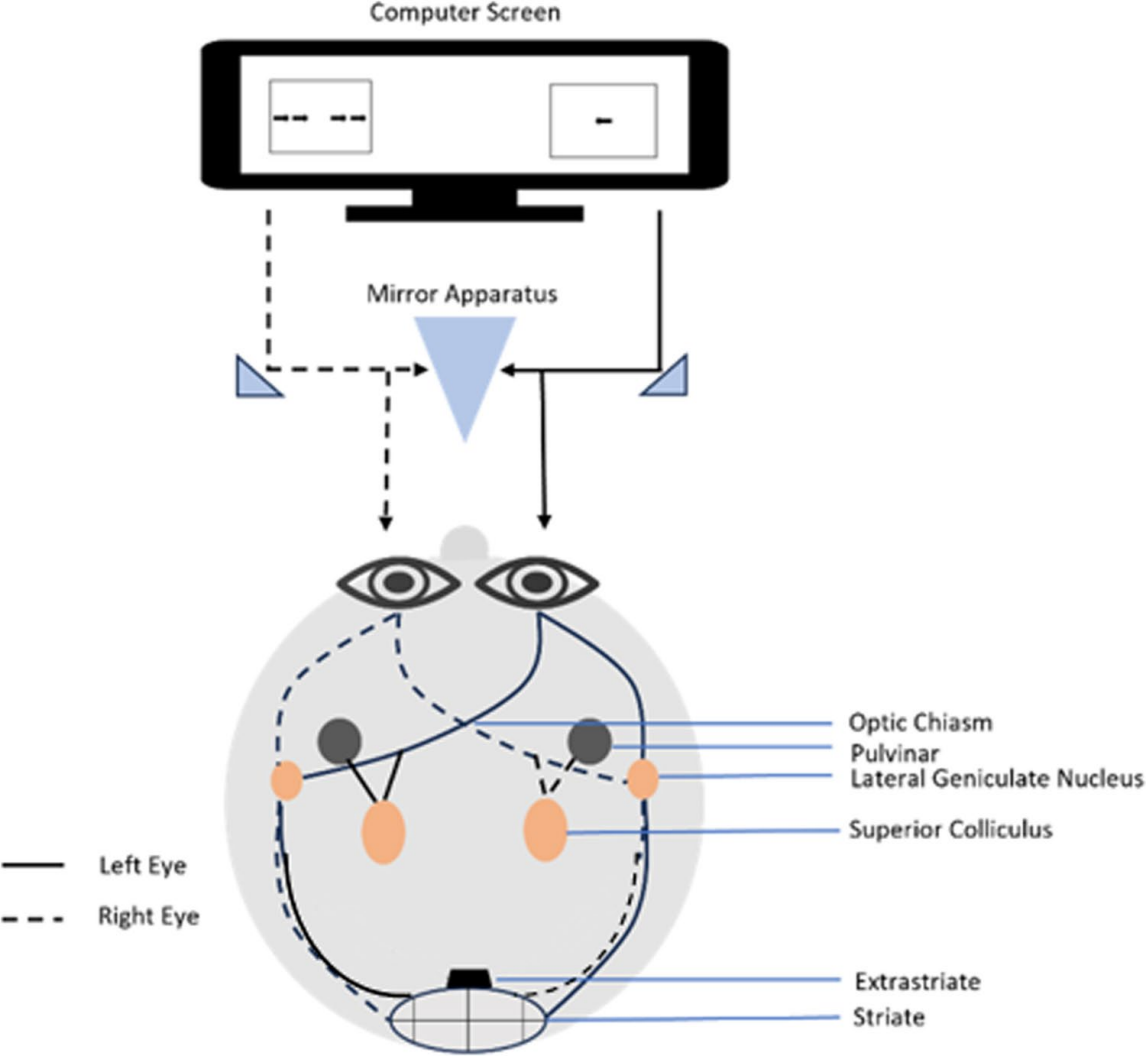
Experimental apparatus and visual pathways from the eyes to the brain. Through the optic nerve, visual information reaches the lateral geniculate nucleus (LGN) of the thalamus. The LGN has axon tracks that terminate predominantly in layer 4 of V1. Each track is sensitive to monocular information, whereas neurons in the striate cortex and cortical regions are primarily sensitive to binocular information

In the experimental paradigm, participants were seated approximately 45 cm in front of a computer screen with a chin rest used to stabilize the head. All stimuli were presented on a white background using a 22” CRT monitor with a 1680 × 1050 screen resolution and a 59-Hz refresh rate. The stimuli were black arrows. Each arrow measured 0.57° high x 1.14° wide and was oriented vertically in the middle of the screen (the distance between each two adjacent arrows was 1.7°). The screen was divided into two parts; each half of the screen was presented to a different eye through the use of a stereoscope (Fig. 1). On each side of the screen, a large rectangle was presented to one eye (and also was used to enhance convergence between the eyes). All visual stimuli were presented within the large rectangular frames.

### Tools

To study the involvement of subcortical regions in executive inhibition task, we employed a stereoscope, thus enabling us to manipulate the visual information presented to each eye separately. This technique allowed us to examine the involvement of monocularly segregated subcortical regions of the visual processing stream (Fig. 1). Stereoscope use relies on the fact that visual input is monocularly segregated until it reaches striate regions (Horton et al., 1990; Menon et al., 1997). Subcortical regions, such as the thalamus, are eye-dependent, whereas cortical regions, such as the extrastriate cortex, are mostly insensitive to the eye-of-origin of the visual information. Through the optic nerve, visual information reaches the lateral geniculate nucleus (LGN) of the thalamus. The LGN has axon tracks that terminate predominantly in layer 4 of V1. Each track is sensitive to monocular information, whereas neurons in extrastriate regions of the visual cortex are primarily sensitive to binocular information (Menon et al., 1997). Presenting different visual information to each eye separately is an effective method for isolating the involvement of monocular (mostly subcortical: thalamic regions) and binocular (mostly cortical: striate cortex) neural channels. Hence, if these areas are functionally involved in executive inhibition processes, dividing the visual information will affect performance on this task. In contrast, if subcortical areas are not involved in those processes (e.g., if they only channel information to cortical areas), segregating the visual information between the eyes will not modulate performance on that specific task. Specifically, in the current study, the computer monitor positioned 57 cm in front of a stereoscope (model ScreenScope LCD SA200LCD), so that the participant's direct view of the monitor is blocked (Fig. 1). Each eye was shown half of the screen presentation.

### Procedure

Before the training block, we conducted two calibration tests to ensure that the participants’ percept was well-fused. First, we asked participants whether they saw a single rectangle or two overlapping rectangles when looking through the stereoscope (note that two rectangles were presented throughout the task, one to each eye, and all stimuli were presented inside those rectangles). If participants reported seeing two overlapping rectangles, the stereoscope was calibrated to achieve a fused percept of a single rectangle. Second, participants were instructed to close one eye (this was done for each eye separately) and asked if they saw a full rectangle (to make sure that the visual display was full for each eye separately). If participants reported seeing only a part of the rectangle, the stereoscope was recalibrated. These tests assured us that the percept was well-fused during the task.

**Fig. 2:**
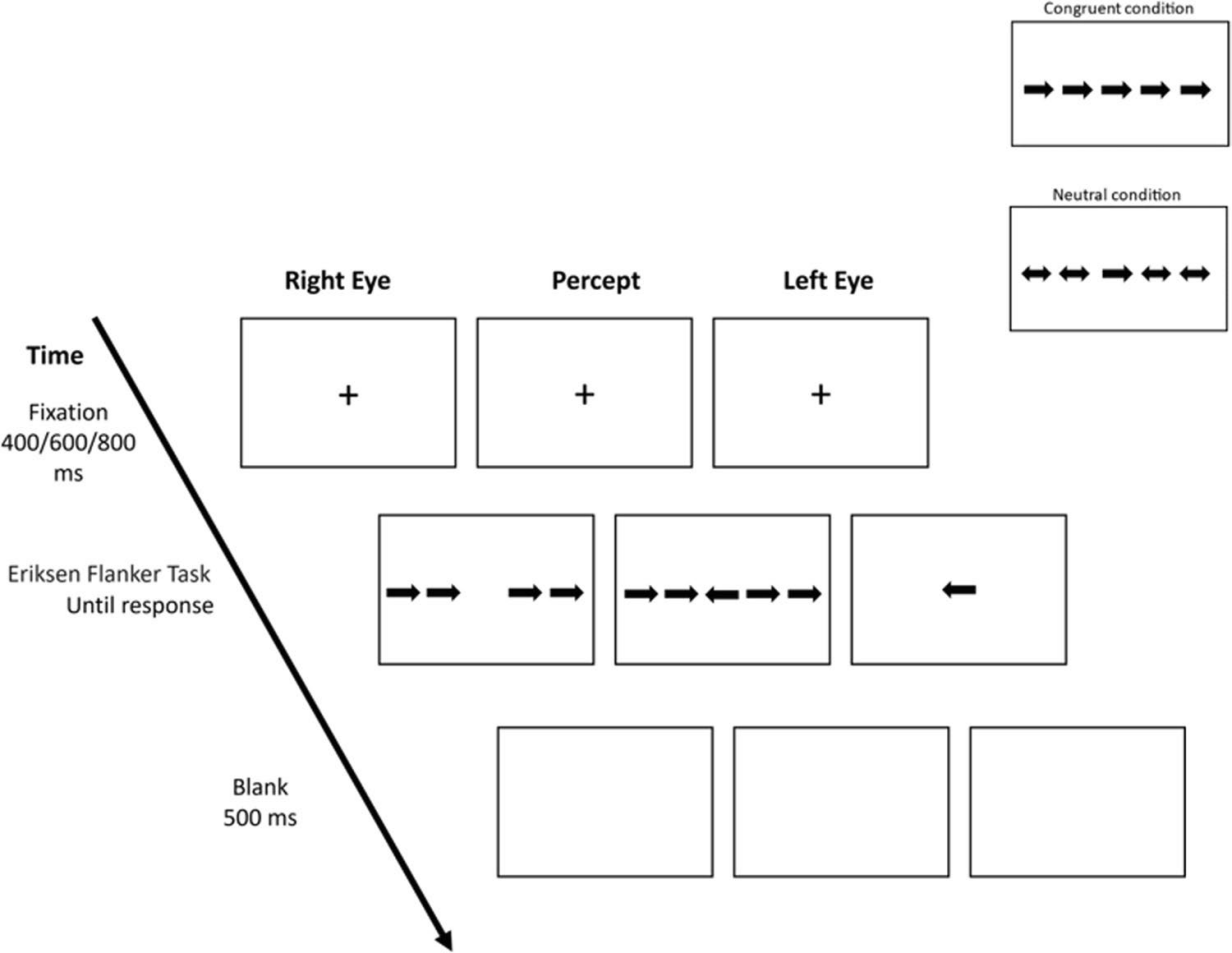
An incongruent condition in a dichoptic eye presentation trial. Dichoptic condition: target arrow is presented to one eye and the flankers to the other eye. The percept is the same as in the eye validity conditions. Congruent and neutral stimuli (right upper)

Each trial started with a fixation period for either 400, 600, or 800 ms, followed by five arrows of which the central one is the target arrow and four flanker arrows surrounding it. The arrows were presented until response (Fig. 2). A blank screen was presented for 500 ms after the participant responded. Participants were instructed to focus on a centrally located fixation cross and maintain their gaze at that location throughout the task. Then, they were asked to respond according to the direction of the target arrow by pressing “Q” if the arrow points left or “P” if it points right, as quickly and accurately as possible. The flankers had three *congruency* conditions: congruent, incongruent, and neutral. In the congruent condition, the central and flanker arrows pointed to the same direction. In the incongruent condition, the central and flanker arrows pointed to opposite directions. In the neutral condition, the flanker arrows were double-headed, meaning that they pointed in both directions. Moreover, by using a stereoscope, we manipulated the *eye presentation* condition; in half of the trials, all the arrows were presented to the same eye, whereas in the other half, the central target arrow was presented to one eye and the flankers to the other eye. There were 36 trials for each of the 6 experimental conditions for a total of 216 trials. There was a practice block with 15 trials followed by 3 experimental blocks with 72 trials. Reaction time (RT) was measured. If inhibition processes involve subcortical regions, in the dichoptic condition, in which the monocular channel that is presented with the target is not presented with the flankers, the flanker distraction effects should be reduced or eliminated compared with the monoptic condition.

## Results

**Fig. 3:**
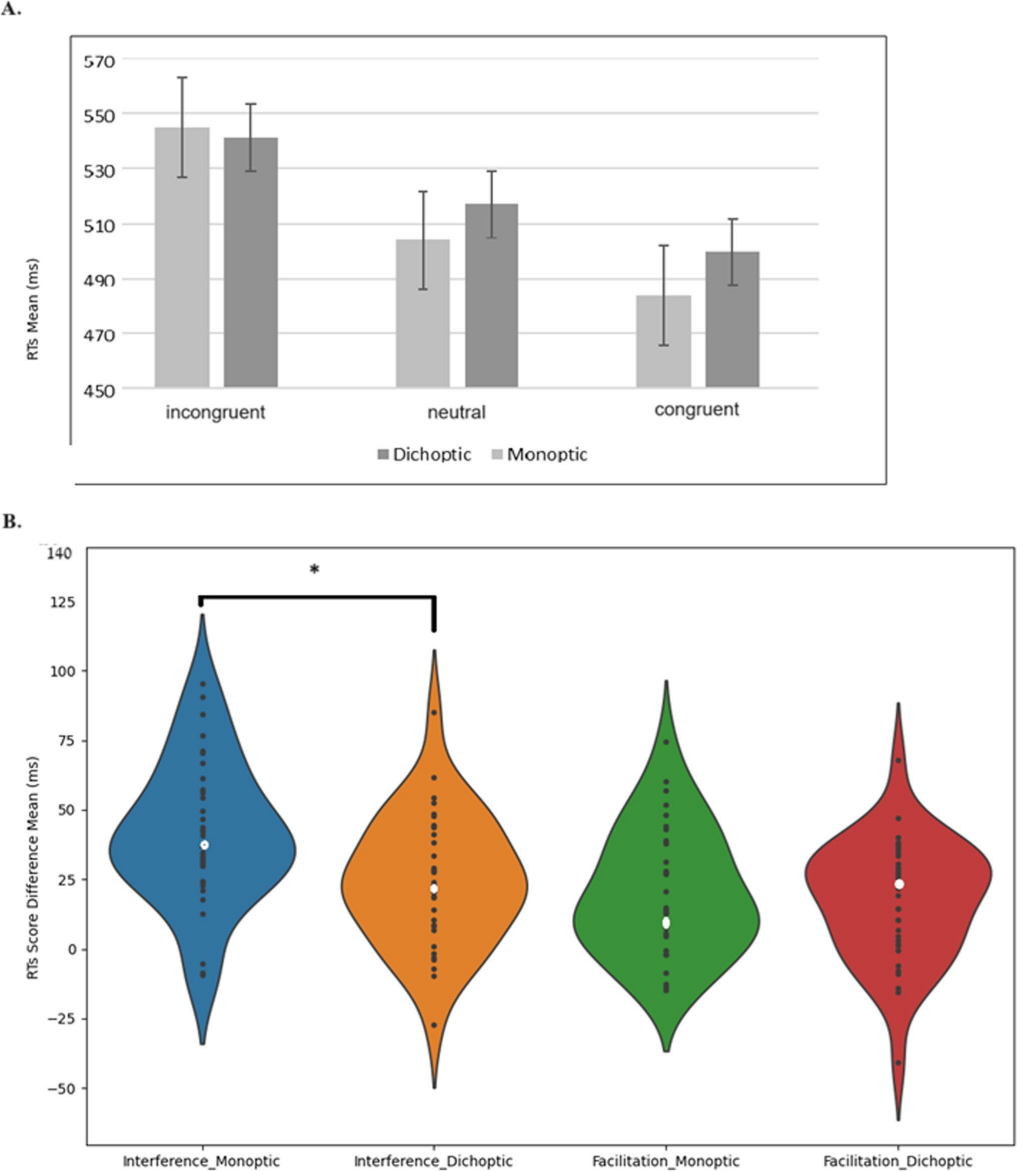
**A.** RTs as a function of congruency and eyes-presentation conditions. Error bars represent 1 standard error from the mean using a method to compute error bars in a within-subject design (Cousineau, 2005). **B.** RT score difference as a function of eye presentation and effect type. The facilitation effect was calculated as mean RTs of neutral trials minus mean RTs of congruent trials. The interference effect was calculated as mean RTs of incongruent trials minus mean RTs of neutral trials. A significant difference in the interference effect between the eye presentation conditions was demonstrated, with greater interference in the monoptic condition compared with the dichoptic condition. There was no difference in the facilitation effect between the two eye presentation conditions. *p < 0.05

Trials in which participants responded incorrectly were discarded (<10%). For each participant, responses in which RTs were faster than 100 ms or slower than 2000 ms also were discarded (4.9%). A two-way analysis of variance (ANOVA) was conducted on RT in the task, with eye presentation (monoptic, dichoptic) and congruency (congruent, neutral, incongruent) as repeated measures factors (Fig. 3A shows all means and standard errors).

There was a significant main effect for congruency [F (2, 70) = 156.51, *p* < .001, ηp^2^ = .81]. Simple effect analyses showed that RTs in the incongruent condition were significantly slower than in the neutral condition [F (1, 35) = 140.94, *p* < .001, ηp^2^ = .77], and RTs in the neutral condition were significantly slower than RTs in the congruent condition [F (1, 35) = 49.14, *p* < .001, ηp^2^ = .56 ]. The main effect of eye presentation was not significant [F (1, 35) = 3.33, *p* = .076, ηp^2^ = .70].

The congruency × eye presentation interaction was significant [F (2, 70) = 6.83, *p* < .01, ηp^2^ = .16].

We further explored the differences in the facilitation effect (i.e., neutral RT > congruent RT) and interference effect (i.e., incongruent RT > neutral RT) between the two presentation conditions. There was no difference in the facilitation effect between the two presentation conditions [F (1, 35) = 0.29, *p* = .590, ηp^2^ = .01]. The interference effect was significantly bigger for the monoptic condition compared with the dichoptic condition [F (1, 35) = 8.23, *p* < .01, ηp^2^ = .19] (Fig. 3B). Further analysis revealed that the facilitation effect was significant both in the monoptic condition [F (1, 35) = 28.93, *p* < .001, ηp^2^ = .96], and in the dichoptic condition [F (1, 35) = 24, *p* < .001, ηp^2^ = .92]. In addition, the interference effect was significant both in the monoptic [F (1, 35) = 94.63, *p* < .001, ηp^2^ = .73], and the dichoptic condition [F(1,35) = 40, *p* < 0.001, ηp^2 ^= .53].

## Discussion

The primary objective of our study was to examine the involvement of subcortical regions in EFs processes, focusing on inhibition abilities. The current experiment investigated the involvement of subcortical monocular visual pathways in executive inhibition processes. The results revealed greater **interference** in the monoptic condition than the dichoptic condition. However, such differences were not observed for the **facilitation effect**. These findings suggest that subcortical regions play a substantial role in inhibitory processes, especially in the representation of the executive conflict (as evidenced by the monoptic involvement in the interference effect). Furthermore, these results imply that while interference is performed by subcortical areas, conflict resolution (facilitation effect) is executed by cortical regions.

This pattern of results aligns with Saban et al.’s (2018) findings. In that study, a Stroop task was used with a stereoscope method to examine the involvement of monocular visual pathways in inhibition and control processes, and specifically in interference and facilitation effects. Their main findings showed a greater interference effect in the monoptic condition compared with the dichoptic condition, whereas there were no differences between the eye presentation conditions in the facilitation effect. Both the Stroop task and the Eriksen flanker task assess executive inhibition and control. Therefore, the current replication validates the role of subcortical regions in the representation and interference of a conflict. Moreover, the results underline the importance of cortical regions in conflict resolution, consistent with previous studies showing that cortical regions play a major role in inhibitory processes (Aron & Poldrack, 2006; Miller & Cohen, 2001).

Furthermore, the current research underscores the importance of using research methods that allow for examination of subcortical involvement to gain a deeper understanding of the neurocognitive systems mediating behavior. This noninvasive approach enables for an in-depth understanding of subcortical mechanisms and their functional roles in various processes and abilities. Previous studies using stereoscopic methodology have yielded noteworthy findings regarding the involvement of subcortical regions across various cognitive domains, including face representation (Gabay et al., 2014), attentional processes (Gabay & Behrmann, 2014; Strommer et al., 2024), visual perception (Soloveichick et al., 2021; Zeng et al., 2024), and executive functions (Saban et al., 2018; Peskin et al., 2020). This collective body of research contributes to a comprehensive understanding of the subcortical structures’ functional role in cognitive processes.

Insights into subcortical mechanisms also can be gleaned from animal studies, which demonstrate executive abilities in the absence of a fully developed cortex. For instance, attentional processes similar to those of humans were demonstrated in archerfish (Gabay et al., 2013; Saban et al, 2018). Archerfish have an optic tectum but lack fully developed cortical structures. In one study (Gabay et al., 2013), archerfish were trained to perform a Posner cuing task (Posner et al., 1980), and demonstrated the typical pattern of results observed in humans: namely, both facilitation of target processing at a cued location during short cue-target delays, and inhibition of return (IOR) to that cued location during longer cue-target delays. These results indicate that space-based attention can be performed without cortical involvement in certain species and, therefore, may rely on both cortical and subcortical visual pathways.

The results of our study hold implications for both research and practical applications. One aspect that deserves attention is the potential impact on individuals with traumatic brain injuries, particularly those with subcortical lesions. Insights into the nuanced interactions between subcortical regions and EFs may inform targeted recovery strategies for such patients. Moreover, our study offers compelling possibilities for clinical applications, particularly in the realm of neurofeedback. This innovative technique has demonstrated efficacy in various contexts, including enhancing cognitive functions (Enriquez-Geppert et al., 2013; Wang & Hsieh, 2013), alleviating symptoms of certain neurological disorders (Marzbani et al., 2016; Anil et al., 2021), and promoting overall well-being (Gruzelier et al., 2014). Neurofeedback involves providing real-time information about brain activity, allowing individuals to learn self-regulation and potentially influence subcortical processes (Hammond, 2007). By further exploring and harnessing the benefits of neurofeedback, researchers and clinicians may uncover valuable therapeutic avenues for improving EFs and cognitive performance in both clinical and nonclinical populations.

The present study encountered certain limitations that warrant consideration. First, the examination of EFs was restricted to one component, inhibition, and its respective measurement task: the Eriksen flanker task. To enhance understanding of the neural mechanisms underlying EFs, it is important to extend this investigation to encompass this component (inhibition) through diverse tasks, such as the go/no-go task, spatial span task, etc. Additionally, the inclusion of more components of EFs, such as shifting and updating, would contribute to a more nuanced analysis. Second, the stereoscope method used in the study facilitated the investigation of subcortical involvement in a noninvasive manner. However, owing to the segregation within the visual pathways, it remains uncertain which specific subcortical regions are involved in these executive processes. Clarifying these aspects would further refine our understanding of the neural substrates associated with EFs.

## Conclusions

The current study provided insights into the complex interplay of subcortical regions in EFs, particularly inhibition processes. The consistent emergence of a monoptic advantage in representation of conflict underscores the substantial contribution of subcortical regions to executive processes. Looking forward, future investigations could extend this line of inquiry by exploring the involvement of subcortical regions in the shifting process, as well as more nuanced conditions of conflict representation and resolution, offering a more comprehensive understanding of the interrelation between different aspects of EFs.

## Data Availability

Will be provide by request.

## Code availability

Will be provide by request.
